# The Effectiveness of Mindfulness-Based Cognitive Therapy in Primary Care and the Role of Depression Severity and Treatment Attendance

**DOI:** 10.1007/s12671-021-01794-3

**Published:** 2021-11-29

**Authors:** Matilde Elices, Víctor Pérez-Sola, Adrián Pérez-Aranda, Francesc Colom, Maria Polo, Luis Miguel Martín-López, Miguel Gárriz

**Affiliations:** 1 Institut de Neuropsiquiatria i Addiccions (INAD), Institut Hospital del Mar d’Investigacions Mèdiques, (IMIM), Parc de Salut Mar, Barcelona, Spain; 2 Centro de Investigación Biomédica en Red de Salud Mental, CIBERSAM, Madrid, Spain; 3 Departament de Psicologia Bàsica, Facultat de Psicologia, Universitat Autònoma de Barcelona (UAB), Edifici B, 08193, Bellaterra, Cerdanyola del Vallès, Barcelona, Spain; 4 Universitat Abat Oliba CEU, Barcelona, Spain

**Keywords:** Mindfulness-based cognitive therapy, Primary care, Effectiveness, Depression, Implementation

## Abstract

**Objectives:**

Evidence suggests the efficacy of mindfulness-based cognitive therapy (MBCT) to prevent depression relapse and decrease depressive symptoms during the acute phase. However, the effectiveness of MBCT in real-world heterogeneous samples treated in clinical health settings, including primary care, has received little attention. This study had two aims: (1) to evaluate the effectiveness of MBCT delivered in primary care considering pre-treatment depression scores and (2) to explore the role of participants’ characteristics on symptom improvement.

**Methods:**

Data were obtained from 433 individuals who received MBCT. Participants completed the Personality Inventory for ICD-11 (PiCD) pretreatment and the Beck Depression Inventory (BDI-II) pre- and post-treatment.

**Results:**

Sixty percent presented moderate-to-severe depression according to scores on the BDI-II, 18.1% presented mild depression, and 21.7% were in the non-depressed range. The severity of pre-treatment depressive symptoms was associated with outcomes. Most individuals who lacked depressive symptoms at baseline remained in the non-clinical range after the treatment. Those in the severe group benefited the most from the intervention, since 35.6% were considered recovered. Rates of deterioration ranged from 2.1 to 2.7%, depending on the depression-baseline scores. Depression severity at the entrance, attendance, and age, but not personality traits, appear to be related to symptom improvement.

**Conclusions:**

According to our results, MBCT can be effectively and safely delivered in primary care.

Mindfulness-based interventions (MBIs) have been increasingly used in healthcare (Kriakous et al., 2021). A variety of MBIs have been developed to treat specific mental health conditions, including substance abuse, depression, anxiety, and eating disorders (Goldberg et al., 2018). Mindfulness-based cognitive therapy (MBCT; Segal et al., 2002) was developed to deal with depressive recurrence. MBCT merges traditional cognitive behavioral therapy (CBT) with mindfulness to provide individuals with a history of depression the skills to detect early signs of mood deterioration and respond to them efficiently. Several RCTs have established the efficacy of MBCT to prevent depression relapse (Kuyken et al., 2015; Ma & Teasdale, 2004; McCartney et al., 2021; Teasdale et al., 2000; Teasdale et al., 2002), showing that MBCT plus treatment as usual (TAU) reduces relapse risk and is as efficacious as maintenance with antidepressant medication (Kuyken et al., 2015). As a result of the accumulated evidence, the UK National Institute for Health and Clinical Excellence recommends MBCT to prevent depression relapse (Abraham et al., 2009).

More recently, research has shifted to explore the efficacy of MBCT in other psychiatric conditions such as bipolar disorder, anxiety disorders, and acute depression (Goldberg et al., 2019; Ives-Deliperi et al., 2013; Mcmanus et al., 2012). While the efficacy of MBCT for these clinical conditions is still growing, studies focused on implementation in different healthcare settings and its effectiveness in real-world samples are scarce. Indeed, it is estimated that less than 1% of mindfulness research is focused on the implementation or effectiveness of MBIs (Dimidjian & Segal, 2015). Collecting data in real-world heath settings, including primary care, is crucial to investigate the effectiveness of psychological interventions beyond RCTs, which results may not be valid to patients treated routinely in community settings (Cuijpers et al., 2019).

Primary healthcare intends to provide universally accessible healthcare to individuals in the community, close as possible to where people live. There are many advantages for delivering mental health services in primary care, including early detection and treatment, improved follow-up, reduced stigma for people with mental disorders, and a better context to treat comorbidities between physical and mental health (WHO and Wonca Working Party on Mental Health, 2008). The integration of mental health into primary care has led to early diagnosis, with studies reporting that individuals treated in primary care often present depression, anxiety, or adjustment disorders (Staab et al., 2001; Sundquist, Ohlsson, et al., 2017). According to recent reviews (Cuijpers et al., 2019; Santoft et al., 2019), psychological therapies such as CBT seem to be effectively delivered in primary care. These treatments appear to effectively reduce symptoms in those experiencing depression and seem to have a preventive effect for those presenting subthreshold depression (Cuijpers et al., 2019; Santoft et al., 2019). There are several reasons why MBCT might offer an advantageous approach to treat depression in primary care: (1) MBCT is considered adequate for patients at different illness stages, including acute depressive symptoms (Goldberg et al., 2019) and partial/complete remission (Kuyken et al., 2016); (2) MBCT is a good alternative for those who do not want or are not able to continue with antidepressant treatments (Kuyken et al., 2015); (3) MBCT is delivered in a group format, potentially reducing costs and the workload of mental health professionals (Feliu-Soler et al., 2017; Saha et al., 2020); and (4) MBCT seems to target some of the mechanisms related to a wide range of psychopathological conditions including worry, rumination, and decreased mindfulness (Alsubaie et al., 2017; van der Velden et al., 2015).

Although data from real-world healthcare settings are scarce, a few exceptions exist. For example, Tickell et al. (2020) collected data from participants receiving MBCT in five mental health services from different regions in the UK. That study showed that almost half of the sample that received MBCT did not reach the clinical cutoff score on the depression questionnaire at pre-treatment. While participants who were not depressed at entry sustained their state, those who entered the depressed range experienced a significant reduction in depression severity. Interestingly, they also report data on reliable deterioration, which was estimated in 3% of the sample (Tickell et al., 2020). These findings are relevant as they demonstrate that MBCT is being used more extensively from its original target population and confirm that MBCT is delivered safely. However, the sample used by Tickell et al. (2020) was treated in mental health–specialized services. Therefore, these findings may not be transferred to the population treated in primary care, which tends to be more heterogeneous (Demarzo et al., 2015).

On the other hand, in Sweden, mindfulness-based group therapy delivered in primary care for individuals with depression, anxiety, and adjustment disorders was compared to individual CBT, finding that both treatments were efficacious in reducing psychiatric symptoms (Sundquist et al., 2015; Sundquist, Palmér, et al., 2017). Recently, it was reported that these beneficial effects were maintained after 12 months (Sundquist et al., 2019). It is worth mentioning that the intervention delivered in these studies was a combination of MBCT and mindfulness-based stress reduction (MBSR; Kabat-Zinn, 1990).

In line with Sundquist’s findings, a previous study (Gárriz et al., 2020) reported that MBCT was effectively delivered for individuals that were treated in primary care and presented a wide range of diagnoses, including adjustment (41.6%), mood (22.7%), and anxiety (14.1%) disorders. MBCT was associated with an improvement of depressive and anxiety symptoms, regardless of the specific diagnosis. Participant’s experiences collected through in-depth interviews revealed a high level of satisfaction with the program, related to the understanding of human distress and suffering and the development of a more present-focused, decentered, and acceptance-based relationship with the experience, which is believed to be a crucial aspect of MBIs (Crane et al., 2017). Participants also pointed out that learning was progressive during the 8 weeks, suggesting a sort of inflection point at session five (Gárriz et al., 2020). This finding is interesting, as it sheds light on the importance of sessions’ attendance as a predictor of outcomes. However, pre-treatment depression symptoms were not considered for the analysis, and therefore, the effectiveness of MBCT for different subgroups of patients remained unknown. In addition, the study did not explore the role of patients’ factors in clinical outcomes and thus lacked data to assist in selecting and optimizing treatment responses for individuals receiving MBCT. Previous research has demonstrated that increased vulnerability associated with childhood trauma was related to a greater benefit from MBCT in comparison with a control intervention (Williams et al., 2014), while other factors such as the amount of home practice (Parsons et al., 2017) or teacher competence (Huijbers et al., 2017) have been related to outcomes but with smaller effects. It has also been suggested that personality dimensions, especially neuroticism, are associated with severity and treatment response in individuals with depression and anxiety disorders (Bagby et al., 2008; Mulder, 2002). However, the relationship between participants’ personality dimensions and symptom reductions resulting from MBIs has been scarcely explored.

The primary aim of this work is to expand existing evidence from other countries, mainly the UK, by providing data on how MBCT is implemented in different regions and settings. The study’s second aim is to explore the impact of participants’ characteristics, including personality traits, on symptom improvement.

## Method

### Participants

Data were collected from a total of 433 individuals who had taken part in MBCT groups between 2018 and 2020. Sixty-seven percent of the sample were women (*n* = 288), and age ranged from 19 to 82 years old (M = 47.73, SD = 11.43). Most of the participants (68.1%) were married or were in a stable relationship and reported finishing high school (67.4%). Almost half of the sample (*n* = 200, 46.2%) were employed. The most common diagnoses in the sample were adjustment disorders (48%), followed by mood disorders (17.8%) and anxiety disorders (11.1%). Two-hundred and seventy-one participants (62.6%) took pharmacological treatment, mostly SSRIs (40.2%). The group receiving pharmacological treatment presented higher BDI-II baseline scores than the group receiving no treatment (*t* = 2.68, *p* = .008) and was more likely to be on sick leave (chi = 15.37, *p* =. 004). According to baseline BDI-II scores, 21.7% were not depressed (i.e., BDI-II scores under 14), 18.1% presented mild depression (i.e., scores between 14 and 19), and 60.3% of service users presented moderate-to-severe depression (i.e., scores above 20). These three subgroups (i.e., no depression, mild depression, and moderate/severe depression) did not differ in sociodemographic characteristics such as age, gender, or marital status. However, participants with moderate/severe depression were less likely to be employed and have university studies, while they were more likely to be on sick leave and presented significantly higher scores in all the personality domains assessed by the PiCD (*p* values ≤ .015) except for anankastic. Table 1 shows the participant’s characteristics at baseline.

**Table 1:** Demographic and clinical baseline characteristics of users participating in mindfulness-based cognitive therapy

	Total sample (*N* = 433)	No depression (*n* = 94)	Mild depression (*n* = 78)	Moderate/severe depression (*n* = 261)	*p* value
Age, M (SD)	47.73 (11.43)	48.16 (11.84)	49.71 (12.10)	46.98 (11.02)	.168
Sex, no. of females (%)	288 (66.5%)	61 (64.9%)	45 (54.9%)	182 (69.7%)	.132
Marital status, *N* (%)					.728
Married/stable relationship	295 (68.1%)	65 (69.1%)	59 (75.6%)	171 (65.5%)	
Single	63 (14.5%)	13 (13.8%)	10 (12.8%)	40 (15.3%)	
Divorced	67 (15.5%)	14 (14.9%)	7 (8.9%)	46 (17.6%)	
Widowed	8 (1.8%)	2 (2.1%)	2 (2.6%)	4 (1.5%)	
Educational level, *N* (%)					.050
Less than high school	17 (3.9%)	2 (2.1%)	1 (1.3%)	14 (5.4%)	
High school graduate	292 (67.4%)	55 (58.5%)	52 (66.7%)	185 (70.9%)	
University graduate	124 (28.6%)	37 (39.4%)	25 (32%)	62 (23.7%)	
Employment status, *N* (%)					< .001
Employed	200 (46.2%)	62 (65.9%)	40 (5.1%)	98 (37.5%)	
Unemployed	61 (14.1%)	9 (9.6%)	12 (15.4%)	40 (15.3%)	
Sick leave	128 (29.6%)	18 (19.1%)	15 (19.2%)	95 (36.4%)	
Disability	21 (4.8%)	1 (1.1%)	2 (2.6%)	18 (6.9%)	
Retired	21 (4.8%)	2 (2.1%)	9 (11.5%)	10 (3.8%)	
Diagnoses, *N* (%)					< .001
Adjustment disorder	208 (48%)	34 (37.8%)	40 (51.3%)	134 (51.3%)	
Mood disorder	77 (17.8%)	7 (7.8%)	14 (17.9%)	56 (21.5%)	
Anxiety disorder	48 (11.1%)	16 (17.8%)	8 (10.3%)	24 (9.2%)	
Personality disorder	12 (2.8%)	1 (1.1%)	1 (1.3%)	10 (3.8%)	
Pharmacological treatment, *N* (%)					.001
No treatment	162 (37.4%)	51 (54.3%)	34 (43.6%)	77 (29.5%)	
SSRI	174 (40.2%)	25 (26.6%)	25 (32.1%)	124 (47.5%)	
Benzodiazepine	46 (10.6%)	9 (9.6%)	9 (11.5%)	28 (10.7%)	
Others	51 (11.8%)	9 (9.6%)	10 (12.8%)	32 (12.3%)	
BDI-II, M (SD)	23.68 (11.80)	8.85 (3.62)	16.38 (1.86)	31.20 (8.57)	< .001
PiCD					
Negative affect	40.94 (7.09)	34.69 (6.90)	38.01 (6.64)	44.00 (6.94)	< .001
Detachment	28.54 (8.50)	23.77 (6.53)	25.91 (6.87)	31.00 (8.63)	< .001
Dissociative	21.94 (5.86)	21.00 (5.06)	20.82 (5.25)	22.61 (6.20)	.015
Disinhibition	24.93 (6.84)	21.69 (5.68)	22.70 (5.57)	26.72 (6.96)	< .001
Anankastic	41.10 (6.51)	41.24 (5.96)	40.79 (5.83)	41.14 (6.89)	.894
Attendance to MBCT sessions, M (SD)	6.18 (2.11)	6.46 (2.11)	5.87 (2.21)	6.17 (2.08)	.193
Attendance to intensive practice retreat, *N* (%)	301 (69.5%)	67 (71.3%)	50 (64.1%)	184 (70.5%)	.723

*M*, mean; *SD*, standard deviation; *SSRI*, selective serotonin reuptake inhibitor; *BDI-II*, Beck Depression Inventory-II; *MBCT*, Mindfulness-Based Cognitive Therapy; *PiCD*, Personality Inventory for ICD-11. PiCD of 414 participants was registered

### Procedures

Participants were recruited through the primary care psychological support program of the Hospital del Mar (Barcelona, Spain). General practitioners (GPs) identified patients that could benefit from psychological assistance and referred them for assessment and treatment. Inclusion criteria for the MBCT program were as follows: (1) age > 18 years, and (2) DSM-5 (American Psychiatric Association 2013) criteria for depressive, anxiety, or stress-related disorders (acute-stress disorder and adjustment disorder). The inclusion of these diagnoses was based on their very high prevalence rates in primary care settings. Diagnoses were based on the judgment of experienced clinical psychologists or psychiatrists. Individuals who met DSM-5 criteria for any of the following disorders were excluded from participation: bipolar disorder, eating disorder, post-traumatic stress disorder, psychotic disorder, borderline personality disorder, substance abuse disorder, and suicidality. Participants presenting either of these diagnoses were referred to specialized mental health services for treatment. A detailed description of the care pathway can be found elsewhere (Gárriz et al., 2020). Within this primary care support program, psychological treatments were delivered in GP practices. Those meeting the eligibility criteria for MBCT and who agreed to participate were scheduled for enrolment in the next available group. Separate groups of 14–18 participants were run simultaneously at different times.

MBCT was delivered by an experienced teacher and mindfulness practitioner (10 years of experience) with a background in clinical psychology and a Ph.D. degree. MBCT was provided in weekly sessions of 2.5 h for 8 weeks, with an extra full day of mindfulness practice (5 h). MBCT was derived from MBSR (Kabat-Zinn, 1990) and CBT for depression and was originally developed to prevent relapses in patients with recurrent depressive disorder (Teasdale et al., 2000). Through the regular practice of different mindfulness exercises and cognitive behavioral skills, patients become more aware of bodily sensations, thoughts, and feelings associated with low mood, consequently being able to relate in a non-attached manner to those experiences and feelings.

### Measures

Sociodemographic characteristics were obtained using an ad hoc questionnaire that was completed before session 1. Clinical diagnoses were collected from the participant’s medical records. The lead psychologist recorded attendance at each MBCT session and the day of intensive mindfulness practice.

The Beck Depression Inventory-II (BDI-II; Sanz et al., 2005) was the primary outcome measure. This 21-item questionnaire assesses the severity of depressive symptoms, including affective, behavioral, cognitive, and somatic symptoms, indicative of unipolar depression. The BDI-II is self-administered, and items are rated on a 0–3 scale; total score ranges from 0 to 63, and higher scores indicate higher severity. The Spanish version has good psychometric properties, including internal consistency (Cronbach’s *α* = 0.89) and convergent and divergent validity. Although traditionally BDI-II scores are divided into four groups (i.e., without current depression, mild, moderate, and severe depression), for this study, three cutoff points were used: 0–13 no depression, 14–19 mild depression, scores above 20 were considered moderate/severe depression.

The Personality Inventory for ICD-11 (PiCD; Oltmanns & Widiger, 2018) is a 60-item self-report measure designed to assess the five broad domains of personality disorder proposed by the World Health Organization’s International Classification of Diseases (ICD-11; Gaebel et al., 2017): (1) negative affect, which is characterized by the tendency to manifest a broad range of distressing emotions (e.g., anxiety, anger, self-loathing, irritability, depression) in response to even relatively minor stressors; (2) detachment, which is primarily characterized by emotional and interpersonal distance, as manifested by marked social withdrawal or indifference to people; (3) dissocial, which refers to the lack of empathy, aggression, ruthlessness, and hostility against others; (4) disinhibition, which includes traits as impulsivity, irresponsibility, and distractibility; and (5) anankastic, which includes perfectionism, deliberativeness, orderliness, and concern with following rules (Tyrer et al., 2015). Each domain is measured by 12 items scored on a 5-point Likert scale (1 = strongly disagree, 5 = strongly agree). The Spanish version of PiCD has been recently validated (Gutiérrez et al., 2020) presenting good internal consistency (*α* = .75 to .84).

### Data Analyses

Sociodemographic characteristics of the sample are reported using the mean (M) and standard deviation (SD) or the frequency and percentage when appropriate. The effects of the intervention were analyzed using the Student *t* test and considering an intention-to-treat perspective (ITT, *N* = 433). Baseline missing values were replaced only when they represented less than 15% of the patients’ self-reported outcome, using the item’s mean score in our sample. Post-treatment missing values were handled using the last observation carried forward (LOCF) method. The overall percentage of missing post-intervention data was 29.8% (*n* = 129). Data were checked for normality and outliers.

To study the characteristics of those patients who showed greater attendance to the MBCT intervention, the chi-squared test and Student’s *t* test were used to assess significant differences between those participants who attended five sessions or more and those who dropped out (i.e., attended 4 sessions or less). A repeated measures ANOVA was conducted to assess potential differences in the effect of the intervention.

#### Treatment Effect on Depressive Symptoms

Pre-post-intervention differences in depressive symptoms (BDI-II scores) were explored using paired *t* tests, and Cohen’s *d* was used for calculating effect sizes. Cohen’s criteria were used for interpreting effect sizes (small ≥ 0.20, medium ≥ 0.50, and large ≥ 0.80). Paired *t* tests were conducted for the whole sample and subgroups of patients according to their baseline BDI-II scores. BDI-II scores above 20 were interpreted as moderate/severe depression, and scores between 14 and 20 were classified as mild depression, while scores below 14 were classified as no depression.

The reliable change index (RCI) and the clinically significative change criterium (CSC) were calculated following the procedures described by Jacobson and Truax (1991). Normative data from non-patients were used (Sanz et al., 2005) to establish if patients achieved improvement or deterioration of symptoms.

The RCI was calculated according to the following formula:

$$\mathrm{RCI}=\frac{X\mathrm{pre}-X\mathrm{post}}{S\ \mathrm{diff}},\mathrm{where}\ S\mathrm{diff}=\sqrt{2\times {\left(\mathrm{SE}\right)}^2,\mathrm{and}\kern0.5em \mathrm{SE}=\mathrm{SD}\times \sqrt{1-r}\ }$$

*X*pre: group mean before intervention
*X*post: group mean after intervention
SE: standard error
SD: standard deviation
*r*: reliability of the measurement (Cronbach’s alpha)

A pre-post-intervention difference of ≥ 10.85 points on the BDI-II was considered a reliable improvement. On the contrary, an individual who presented a pre-post-intervention increase of ≥ 10.85 points on the BDI-II was considered reliably deteriorated.

For the individuals who presented a lack of depressive symptoms at the entrance, sustained recovery was reported if they maintained BDI-II scores under the cutoff point at post-treatment. Individuals in the clinical groups (i.e., mild or moderate/severe depressive symptoms) were considered recovered if they showed both a reliable change (pre-post-intervention a reduction of 10.85 points) and a clinically significant change.

$$\mathrm{CSC}=\frac{\left(\mathrm{SD}\ \mathrm{normative}\ \mathrm{data}\right)\left( Xp\mathrm{atients}\right)+\left(\mathrm{SD}\mathrm{patients}\right)\left(X\mathrm{normative}\ \mathrm{data}\right)}{\mathrm{SDnormative}\ \mathrm{data}+\mathrm{SDpatients}}$$

Using the formula, the cutoff point was 15.04. Therefore, patients who scored below those after intervention were considered remitted.

#### Predictors of Depressive Symptom Change

Linear regressions and logistic regression models were computed to analyze the predictive role of sociodemographic (i.e., sex, gender, level of education, marital status, and employment situation), clinical-related variables (i.e., baseline level depression and personality domains as measured by the PiCD), and intervention-related outcomes (i.e., number of sessions attended and having abandoned the intervention) on the outcomes of the MBCT program (i.e., changes in the raw scores of BDI-II, number of reliable improvements and deteriorations, and number of remitters).

## Results

### Participants’ Attendance

On average, participants attended 6.18 (SD = 2.11) MBCT sessions, and 82 participants (18.9%) dropped out (attended four sessions or less). Most of the sample (69.5%) participated in the intensive practice retreat day. In contrast to those that attended five or more sessions, participants who dropped out presented significantly lower scores in anankastic personality (*p* = .008). These groups (i.e., low and high attendance) did not significantly differ on any other personality trait. Attendance did not differ between those with moderate/severe depressive symptoms and those with mild or no depressive symptoms at pre-treatment (*p* = .143). The repeated measures analysis indicated that those who attended 5 sessions or more achieved a significantly greater reduction in BDI-II scores post-treatment compared to those who attended 4 sessions or less (*F* = 33.77, *p* < .001). This analysis was conducted after controlling for anankastic personality scores in the PiCD. Moreover, a significantly greater proportion of patients achieved a RCI post-treatment among those who attended 5 or more sessions (29.6% vs. 0%; *χ*^2^ = 36.58, *p* < .001).

### Treatment Impact on Depressive Symptoms

Considering the total sample (*N* = 433, irrespective of BDI-II scores at baseline), mean BDI-II score was 23.68 (SD = 11.80) at pre-treatment and reduced to 18.18 (SD = 12.56) after the intervention, resulting in a statistically significant change (*t* = 11.49, *p* < .001) with a small effect size (*d* = 0.45). In this total sample, 104 patients (24%) achieved a reduction of 10.85 points or more in the total BDI-II score, achieving a reliable improvement. Only 11 users (2.5%) experienced a reliable deterioration.

Most individuals (*n* = 82, 87.2%) who lacked depressive symptoms at baseline (i.e., BDI-II baseline scores < 14) remained in the non-clinical range after the treatment, therefore showing sustained recovery. This subgroup experienced a small significant improvement in residual depressive symptoms (*t* = 2.73, *p* = .008, *d* < 0.29) and 2 participants (2.1%) showed a significant deterioration after the intervention.

Users that began the treatment with mild depressive symptoms showed a significant reduction of BDI-II scores after MBCT (*t* = 4.91, *p* < .001), with a medium effect size (*d* = 0.74). Twenty individuals (25.6%) were recovered considering the CSC (i.e., post-treatment BDI-II score < 15.04), and 11 patients (14.1%) experienced a reliable improvement. On the other hand, two participants (2.6%) showed a reliable deterioration.

Finally, patients who entered the treatment with moderate-to-severe depression scores (BDI-II scores ≥ 20) experienced a significant improvement in depressive symptoms (*t* = 11.27, *p* < .001), this time with a medium effect size (*d* = 0.70). In this case, 93 patients (35.6%) experienced a reliable improvement, and 77 (29.5%) were considered recovered after treatment according to the CSC index. Only seven (2.7%) showed reliable deterioration.

**Fig. 1:**
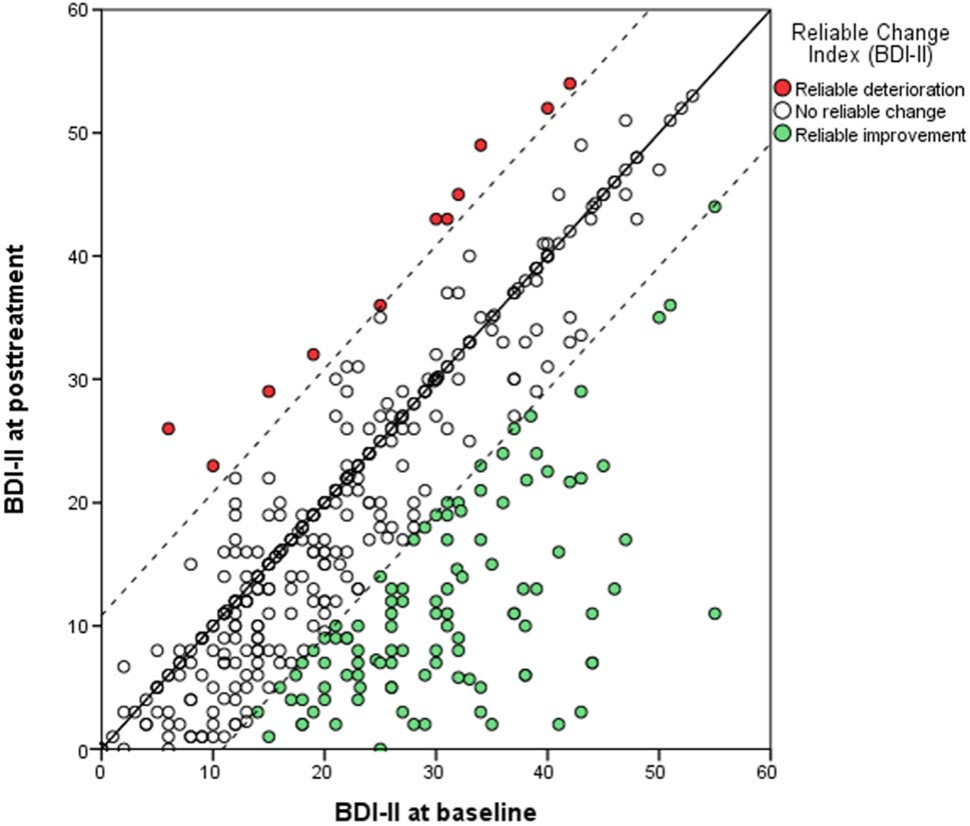
Difference between pre- and post-treatment scores on the Beck Depression Inventory-II (BDI-II) per participant

Table 2 shows mean pre-intervention and post-intervention depression symptoms on the BDI-II for the total sample and each subgroup of participants according to the level of depressive symptoms at baseline. Fig. 1 presents the pre-post-treatment difference for every participant in the sample.

**Table 2:** Mean pre-intervention and post-intervention depressive symptoms for the total sample and each subgroup

	Pre-treatment	Post-treatment	Student’s *t* test (*p*)	Cohen’s *d*
BDI-II, M (SD)				
No depression	8.85 (3.62)	7.46 (5.64)	2.73 (.008)	0.29
Mild depression	16.38 (1.86)	13.13 (5.91)	4.91 (< .001)	0.74
Moderate/Severe depression	31.20 (8.57)	23.56 (12.73)	11.27 (< .001)	0.70
Total sample	23.68 (11.80)	18.18 (12.56)	11.99 (< .001)	0.45

*BDI-II*, Beck Depression Inventory-II

### Factors Related to Clinical Change

Linear regression analyses were computed to estimate the predictive role of different sociodemographic and clinical-related variables on changes in BDI-II scores. Regression models were computed for the total sample, and the groups that showed depressive symptoms. In the total sample, a significant model (*F* = 36.83, *R*^2^ = 0.21*, p* < .001) showed that the reduction of BDI-II scores after the intervention was predicted by baseline level of depressive symptoms (*B* = 0.27, *t* = 7.55, *p* > .001), number of sessions attended (*B* = 1.43, *t* = 7.12, *p* > .001), and age (*B* = −0.11, *t* = −2.81, *p* = .005). This analysis was replicated for the mild and moderate/severe depression subgroups. The same predictive variables were found for the non-depressed subgroup (*F* = 16.89, *R*^2^ = 0.16, *p* < .001). The amount of sessions attended (*B* = 1.06, *t* = 3.60, *p =* .001) was the only significant predictor of changes in BDI-II scores for those with mild symptoms at entrance (*F* = 12.99, *R*^2^ = 0.15, *p =* .001).

## Discussion

Effective psychological treatments are often conceived for specific disorders or diagnostic categories, which constitutes an important barrier to implementing these treatments in real-world settings, with diverse and transdiagnostic populations. In this context, conducting studies with real-world samples is crucial for at least two reasons: first, to shorten the implementation gap by providing treatments that have proven their efficacy in RCTs; and second, to understand how these interventions work in these contexts.

The present study replicates previous works on the efficacy of MBCT, which were mainly conducted in the UK, using a large sample of Spanish patients, and includes new variables such as personality traits as measured by the PiCD. Our results support that MBCT can be effectively and safely delivered in primary care. Regardless of having a clinical depression diagnosis, a large proportion of the sample presented significant subjective moderate-severe depressive symptoms. The severity of pre-treatment depressive symptoms was associated with outcomes. Most individuals who lacked depressive symptoms at baseline remained in the non-clinical range after the treatment, showing sustained recovery. In contrast, those in the severe group benefited the most from the intervention, as suggested by the highest rates of reliable improvement. Rates of deterioration ranged from 2.1 to 2.7%, depending on the depression-baseline scores. Depression severity at the entrance, attendance, and age, but not personality traits, appear to be related to symptom improvement.

Initially, the BDI-II provides four cutoff scores rather than three, differentiating a group with moderate depressive symptoms (scores between 20 and 28) and a group with severe symptoms (scores above 29). Here, we merged the last two groups to create a “moderate-severe” category (scores above 20). Although this decision might be debatable, we considered that working with three groups instead of four provided a more parsimonious model for our data. Using these three categories, we identified three groups: (1) no depression (21.7%), (2) mild depression (18.1%), and (3) moderate-severe depression, representing 60.3% of the sample. Tickell et al. (2020) used the PHQ-9 to characterize their sample into two groups (depression vs. no depression). Even though Tickell used a different scale, the proportion of participants below the clinical cutoff is similar in both studies, showing that MBCT is delivered for a broader population in real-world settings. As expected, the proportion of participants showing a reliable improvement was higher in the moderate-severe group, confirming that MBCT is effective for users presenting acute depressive symptoms (Goldberg et al., 2019; Thimm & Johnsen, 2020). Medium effect sizes (Cohen’s *d* ≥ 0.70) were obtained for symptom improvement in two groups: (1) mild depressive symptoms and (2) moderate-severe depressive symptoms. Although Tickell et al. (2020) reported larger effect sizes (Cohen’s *d* = 0.86), the rates of reliable improvement among those in the most severe group are comparable in both studies. One possible explanation for this is that participants in the previous study (Tickell et al., 2020) may have presented a more severe profile at intake, thus resulting in larger effect sizes.

Although the moderate-severe group differed regarding personality traits, employment status, and education level, no significant differences were found regarding attendance between the three groups. Nineteen percent of the participants dropped out (attended four sessions or less), being in line with previously reported data from RCTs (Kuyken et al., 2015, 2016; Segal et al., 2010) and naturalistic studies (Tickell et al., 2020).

Rates of deterioration in this study were similar to the rates reported in the studies conducted by Tickell et al. (2020) and Baer et al. (2021). As mindfulness-based approaches expand and are offered in community settings, understanding and measuring mindfulness-related adverse events are crucial (Baer et al., 2019). Data collected so far is promising, as studies indicate that mindfulness-based approaches do not increase harm, irrespective of the operationalization of harm, harm domain (physical or psychological), and context (Hirshberg et al., 2020).

In line with Cillessen et al. (2020), our results highlight the importance of session attendance, suggesting that those who attend more sessions achieved a more considerable reduction in depressive symptoms. So far, personality traits as predictors of mindfulness outcomes had been less considered. Findings from recent studies seem to suggest that trait consciousness is related to meditation and program adherence (Canby et al., 2021; Cillessen et al., 2020). However, this is the first time that the relation between personality traits as measured by the PiCD and treatment outcomes of MBCT is explored. Individuals in the moderate-to-severe depression group presented significantly higher scores in almost all traits measured by the PiCD. The association between personality traits, depression diagnosis, and symptoms’ severity has already been described using other personality models, for instance, the big five (Hayward et al., 2013; Koorevaar et al., 2013). According to our findings, personality traits were not related to symptom improvement despite the initial differences between groups. Interestingly, a significant association was found between anankastic personality and attendance, with individuals with lower anankastic traits presenting higher drop-out rates. In line with this finding, previous studies have reported that patients with obsessive traits might show greater adherence and positive attitudes towards psychological treatments than patients with other personality traits (Holma et al., 2010).

Many patients requiring psychological assistance do not fit into a specific diagnosis, presenting mild forms of mood disorders, including anxiety and depressive symptoms often associated with stressful life events (Gárriz et al., 2020; O’donnell et al., 2019). Having data in real-world community samples is crucial to understand whether the treatments being delivered are adequate for the populations who are receiving them. Our findings support the use of MBCT for treating heterogeneous samples in primary care, demonstrating that it is effective and safe. These findings also show that it is possible to shorten the gap between research and routine clinical practice by implementing effective treatments into real-world contexts.

### Limitations and Future Research

First, it needs to be borne in mind that the present study focused on analyzing the effects of MBCT on a range of diagnoses, although most of the sample (almost 50%) presented adjustment disorders. One of the main limitations of the current investigation is the lack of a control group. Without this, it is not possible to ensure that these improvements are attributable to the intervention. The absence of long follow-up periods constitutes another limitation, as longer follow-up would be needed to determine if the benefits gained persist through time. In addition, longer follow-up periods would also be needed to establish if those who entered the program with no-or-mild depressive symptoms can sustain positive mental health. Another limitation is the lack of previous research using the PiCD, hindering our findings’ generalization. Our study also aimed to understand “who benefits” from MBCT; besides the studied variables, other aspects such as the amount of home practice and group-related factors could be related to a differential response to MBCT. Other implementation-related variables (e.g., acceptability, feasibility) should be analyzed as potential predictors of treatment effect in future studies. In addition, the measures used to assess symptoms could have been complemented with other measures such as mindfulness skills or quality of life.

The data reported here is pre-pandemic. As a result of the COVID-19 pandemic, rates of anxiety, depression, and stress-related symptoms among the general population have risen (Xiong et al., 2020), challenging mental health systems and demanding more significant resources to diminish the negative impact on mental health. Without detracting from the aforementioned limitations, our findings support the use of MBCT in heterogeneous populations. A new adaptation of MBCT, called “MBCT for Life,” has been recently developed to reach a broader population that might benefit from mindfulness practice (Strauss et al., 2021). Future studies are needed to determine if MBCT for Life could be more effective than the standard MBCT program for the primary care population.

## Data Availability

The data that support the findings of this study are available from the corresponding author upon reasonable request.
